# To the Editors of the Medical and Physical Journal

**Published:** 1801-12

**Authors:** Walter Scott

**Affiliations:** Hull


### To the Editors of the Medical and Phyſical Journal.

GENTLEMEN,

IF the following Caſes of the ſucceſsful treatment of the ſuppreſſion of urine, by the application of bliſters, &c. above the pubis, meet with your approbation, they are much at your ſervice, and the inſertion of them will confer an honour upon,

Your's, &c.

*Hull, Nov.* 9, 1801. WALTER SCOTT, M. D.

---

A ſoldier, of the Northumberland Regiment of Militia, having contracted a gonorrhœa, did not wiſh to be put upon the ſick report, but very imprudently made uſe of an aſtringent injection; the conſequence of which was, that he was ſeized with a ſuppreſſion of urine, attended with moſt excruciating pain; he was attacked when on duty. It unfortunately happened that I was attending the regiment at exerciſe, which was ſome diſtance from the town; the ſoldier wiſhing for immediate relief, made application to a ſurgeon, a very intelligent young gentleman, who, with the beſt intention in the world, attempted to introduce the catheter, but in this he was foiled, at leaſt no water was drawn off.

The ſoldier was taken to the hoſpital, in the moſt excruciating pain you can poſſibly conceive; I immediately ordered a clyſter to be adminiſtered, and two grains of pure opium to be given by the mouth; blood-letting, fomentations, warm bath, clyſters, laxatives, diuretics, opiates, &c. &c. (cold water alſo was daſhed upon him) were perſevered in for a conſiderable time, viz. for forty-eight hours, without the leaſt relief; clyſters now could not be adminiſtered, from the vaſt preſſure made upon the rectum by the diſtenſion of the veſica urinaria. The patient having received no relief, I ordered a large bliſter to be applied above the pubis, which relieved him from the moſt painful diſtreſſing ſituation I ever met with.

It was my intention, after the inflammation had been removed, to have again attempted the introduction of the catheter, and if I had not ſucceeded, rather than the poor ſoldier ſhould not have been relieved, I was determined to have punctured the bladder.

---

I was conſulted, when I was at Ipſwich, by my ingenious friend, Mr. Freer, ſurgeon of the Leiceſter Militia, in a caſe of ſuppreſſion of urine; the methods generally made uſe of had been been put in practice, but without fuccefs. I recommended the immediate application of a blifter above the pubis; Mr. Freer was perfectly agreeable; but, from the idea I had formed of the manner in which the blifter acted, Mr. Freer, inftead of applying a blifter, ordered an embrocation to the part of aq. ammo.; it anfwered the intention, the foldier in a fhort time was relieved.

Since I came to Hull I have had under my care two cafes of fuppreffion of urine, which I removed by the following application: aq. ammo. ol. terebinth. aa. ℥j. M.

I am of opinion, that in cafes of fuppreffion of urine, from fpafm, or in cyftitis, that blifters will be found of very confiderable utility; at the fame time, clyfters, blood-letting, opiates, &c. &c. are not to be omitted.

In thofe cafes I am againft the hafty introduction of the catheter, until the inflammatory fymptoms are removed. I have in feveral cafes of nephritis, applied blifters to the region of the kidnies, with confiderable relief and advantage.

